# Wnt/ß-catenin-mediated p53 suppression is indispensable for osteogenesis of mesenchymal progenitor cells

**DOI:** 10.1038/s41419-021-03758-w

**Published:** 2021-05-21

**Authors:** Xin Zhou, Allyson Beilter, Zhaohui Xu, Ruli Gao, Shunbin Xiong, Adriana Paulucci-Holthauzen, Guillermina Lozano, Benoit de Crombrugghe, Richard Gorlick

**Affiliations:** 1 Division of Pediatrics, University of Texas MD Anderson Cancer Center, Houston, TX 77030 USA; 2 Thomas Scientific, Swedesboro, NJ USA; 3 Houston Methodist Research Institute, Houston, TX USA; 4 Department of Genetics, University of Texas MD Anderson Cancer Center, Houston, TX 77030 USA

**Keywords:** Developmental biology, Bone development

## Abstract

The developmental origins of mesenchymal progenitor cells (MPCs) and molecular machineries regulating their fate and differentiation are far from defined owing to their complexity. Osteoblasts and adipocytes are descended from common MPCs. Their fates are collectively determined by an orchestra of pathways in response to physiological and external cues. The canonical Wnt pathway signals MPCs to commit to osteogenic differentiation at the expense of adipogenic fate. In contrast to ß-catenin, p53’s anti-osteogenic function is much less understood. Both activities are thought to be achieved through targeting *Runx2* and/or Osterix (*Osx, Sp7*) transcription. Precisely, how Osx activity is dictated by ß-catenin or p53 is not clarified and represents a knowledge gap that, until now, has largely been taken for granted. Using conditional lineage-tracing mice, we demonstrated that chondrocytes gave rise to a sizable fraction of MPCs, which served as progenitors of chondrocyte-derived osteoblasts (Chon-ob). Wnt/ß-catenin activity was only required at the stage of chondrocyte-derived mesenchymal progenitor (C-MPC) to Chon-ob differentiation. ß-catenin^–^ C-MPCs lost osteogenic ability and favored adipogenesis. Mechanistically, we discovered that p53 activity was elevated in ß-catenin^–^ MPCs including ß-catenin^–^ C-MPCs and deleting p53 from the ß-catenin^–^ MPCs fully restored osteogenesis. While high levels of p53 were present in the nuclei of ß-catenin^–^ MPCs, Osx was confined to the cytoplasm, implying a mechanism that did not involve direct p53-Osx interaction. Furthermore, we found that p53’s anti-osteogenic activity was dependent on its DNA-binding ability. Our findings identify chondrocytes as an additional source for MPCs and indicate that Wnt/ß-catenin discretely regulates chondrocyte to C-MPC and the subsequent C-MPC to osteoblast developments. Most of all we unveil a previously unrecognized functional link between ß-catenin and p53, placing p53’s negative role in the context of Wnt/ß-catenin signaling-induced MPC osteogenic differentiation.

## Introduction

Endochondral bone formation occurs through a cartilage to bone conversion process, during which cartilaginous tissue serves both as a template for ossification and as an innate source of osteoblasts^1–6^. The cellular means by which a fully differentiated chondrocyte gains the plasticity to evolve into a mature osteoblast, as well as what signaling pathways govern this event, remains elusive.

Canonical Wnt signaling plays diverse roles at different stages of bone development and growth^5–10^. In *Osx*-expressing MPCS, Wnt/ß-catenin plays a switch role between osteogenic and adipogenic fates. Despite the lack of convincing evidence^7^, it is currently accepted that ß-catenin promotes osteogenesis through activating *Runx2* and/or *Osx* transcription^11–13^.

p53 is a well-established tumor suppressor. It is also a vital regulator of cell fate and differentiation^14^. The precise functions and regulatory mechanisms of p53’s physiological roles remain much less understood and appreciated. In limited reports, crosstalk between p53 and Wnt/ß-catenin signaling has been shown to play various roles in a context-dependent manner, such as in smooth muscle cells^15^ and in embryonic stem cells^16^.

p53 exhibits osteo-inhibitory activity in various mouse models^17,18^ Nonetheless, p53 downstream molecular events leading to osteogenic inhibition are not yet defined. p53 null marrow mesenchymal stem cells are more osteogenic and display no apparent difference in their adipogenic and chondrogenic capacities^19,20^. One study shows that p53 inhibits osteoblastic differentiation through microRNA-34-mediated *Runx2* suppression^21,22^. To date, the physiological context of this inhibitory function remains entirely elusive.

Here we used Collagen X (*Col10a1*) and Aggrecan (*Agc1*)-driven ß-catenin conditional lineage-tracing mice to delineate how Wnt/ß-catenin regulates chondrocyte to osteoblast reprogramming. We showed that chondrocytes evolved into osteoblasts through at least two steps, which were differentially regulated by Wnt/ß-catenin. Mechanistically, we discovered that the ß-catenin-deficient MPCS acquired elevated p53 activity and their defect in osteogenic capacity was fully reinstated by merely deleting p53 from them, indicating that Wnt/ß-catenin promotes osteogenesis via a p53 suppression-dependent mechanism.

## Results

### Characterizations of chondrocyte Cre-mediated β-catenin mutant mice reveal an inverse correlation between the trabecular volumes and the numbers of chondrocyte-derived stromal cells

To acquire a mechanistic understanding of the Wnt/β-catenin action initiated from chondrocytes, we generated chondrocyte-lineage-tracing mouse models containing either deleted or stabilized ß-catenin alleles, and systematically quantified and compared the reporter-expressing cells categorized by location and association with bone matrix.

**Fig. 1:**
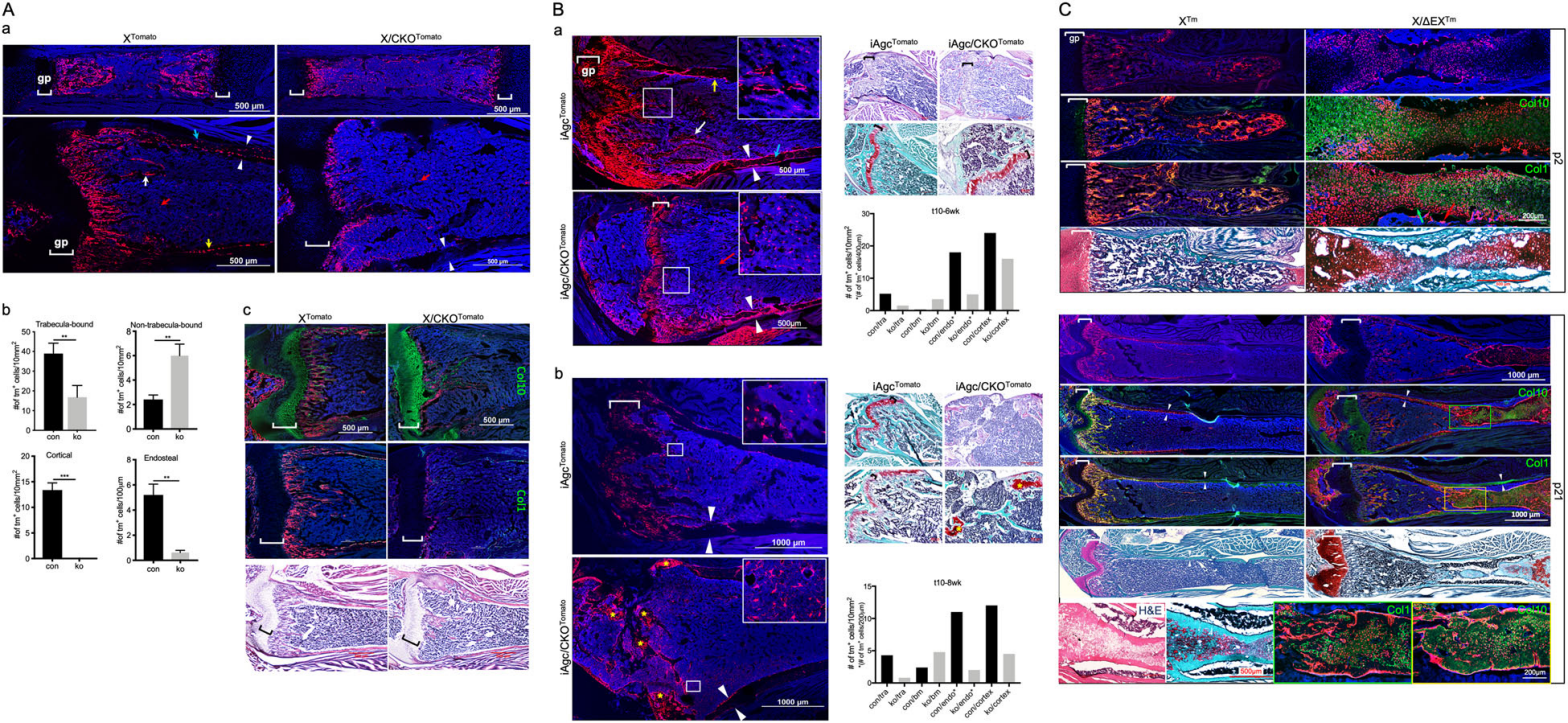
Histological characterization of X/CKO^Tomato^, iAgc/CKO^Tomato^, and X/ΔEX^Tomato^ mice. **A**: **a** Confocal images of the p2 (upper) and p16 (lower) femurs. **b** Quantitative comparisons of Cre-induced Tm^+^ cells categorized by distribution between p16 X^Tomato^ (con) and X/CKO^Tomato^ (ko) mice, *n* = 3, **p < 0.01, ***p < 0.001. **c** Confocal images of p16 femurs: anti-Col10 and anti-Col1 IF (green) and H&E staining. gp growth plate. White arrow: trabecula-bound Tm^+^ cell; red arrow: non-trabecula-bound Tm^+^ cell; blue arrow: cortical Tm^+^ cell; yellow arrow: endosteal Tm^+^ cell. White arrowheads designate cortices. **B** p10 iAgc/CKO^Tomato^ mutant and iAgc^Tomato^ control mice were injected with tamoxifen and sacrificed after 6 weeks (**a**) and 8 weeks (**b**). **a**, **b** Left: confocal images of the femurs. Right top: H&E (upper) and Saf-O (lower) staining; **b** Right bottom: quantitative comparisons of Cre-induced Tm^+^ cells between iAgc^Tomato^ (con) and iAgc/CKO^Tomato^ (ko) mice. Yellow asterisk: cartilage**. C** Confocal and Saf-O images of the p2 humeri and p21 femurs of X^Tomato^ and X/ΔEX^Tomato^ mice. Boxed areas are shown in corresponding magnified images below. Green arrow: indicating Col1 staining lining the mineralized rod. Red arrow: indicting Tm^+^ Chon-obs adjacent to the rod. White arrowheads: denoting cortices.

In the femurs of postnatal animals, an abundant number of Tomato-expressing (Tm^+^) cells was observed within the marrow cavity of both *Col10a1-Cre*;*ROSA26R-Tomato* (X^Tomato^) control and *Col10a1-Cre*;*Ctnnb1*^*fl/fl*^;*ROSA26R-Tomato* (X/CKO^Tomato^) mutant animals, each of which presented a distinct pattern of distribution. At postnatal day 2 (p2) of X^Tomato^ control animal, the majority of *Col10a1-Cre*-induced Tm^+^ (X/Tm^+^) cells were localized at the primary spongiosa physically in contact with trabeculae (trabecula-bound) and showed mature osteoblast-like morphology (Fig. 1Aa). In contrast, most of the Tm^+^ (XCKO/Tm^+^) cells in the p2 X/CKO^Tomato^ mutant animal were scattered rather evenly throughout the marrow cavity. Most of them were not connected with trabeculae (non-trabecula-bound) and were morphologically distinct from mature osteoblasts (Fig. 1Aa). Similarly, at p16, there were more non-trabecula-bound Tm^+^ cells in the X/CKO^Tomato^ mutant than in the X^Tomato^ control mice (6/ko vs 2.4/con cells/10 mm^2^), whereas fewer XCKO/Tm^+^ cells were found on the endostea (0.63/ko vs 6/con cells/100 µm), embedded within cortices (0/ko vs 13.4/con cells/10 mm^2^) or trabecular matrixes (16.6/ko vs 38.9/con cells/10 mm^2^) compared to the X/Tm^+^ cells (Fig. 1Ab). This phenotype persisted and became progressively pronounced with age. In the marrow of 4- and 8-month-old X/CKO^Tomato^ mice, there were visibly more non-trabecula-bound XCKO/Tm^+^ cells than in that of p16, whose non-trabecula-bound XCKO/Tm+ cells remained scattered among the stromal cells and were not embedded within the cortexes (Supplementary Fig. S1a, b).

To validate, we generated *Agc1-CreERT2*;*ROSA26R-Tomato (*iAgc^Tomato^) and *Agc1-CreERT2*;*Ctnnb1*^*fl/fl*^;*ROSA26R-Tomato* (iAgc/CKO^Tomato^) mice. These mice were analyzed after 6- and 8-week chases post tamoxifen injection at p10. The iAgc/CKO^Tomato^ mice developed a low trabecular bone volume phenotype resembling that of the X/CKO^Tomato^ mice (Fig. 1B). After the 6-week chase, numbers of non-trabecula-bound iAgcCKO/Tm^+^ cells were substantially higher than that of non-trabecula-bound iAgc/Tm^+^ cells (3.5/ko vs 0.3/con cells/10 mm^2^). Conversely, numbers of the trabecula-bound, endosteum, and cortex-embedded iAgcCKO/Tm^+^ cells were all fewer than that of the corresponding iAgc/Tm^+^ cells (trabecula: 1.5/ko vs 5.2/con cells/10 mm^2^; endosteum: 5/ko vs 8/con cells/400 µm; cortex: 16/ko vs 24/con cells/10 mm^2^) (Fig. 1Ba). The inverted correlation was preserved after the 8-week chase (non-trabecula-bound: 4.8/ko vs 2.4/con cells/10 mm^2^; trabecula-bound: 0.8/ko vs 4.8/con cells/10 mm^2^; endosteum: 2/ko vs 11/con cells/200 µm; cortex: 4.5/ko vs 12/con cells/10 mm^2^) (Fig. 1Bb). The phenotype gradually intensified with prolonged chases (Supplementary Fig. S2a–c).

In contrast, the *Col10a1-Cre*;*Ctnnb1EX3*^*fl/fl*^;*ROSA26R-Tomato* (X/ΔEX^Tomato^) mice with stabilized β-catenin showed delayed primary ossification and developed an osteopetrosis-like phenotype after birth, opposite of the X/CKO^Tomato^ mice (Fig. 1C and Supplementary Fig. S3a–c). In the p2 X/ΔEX^Tomato^ animal, the humeral cavity was occupied by a rod-shaped structure in place of the primary ossification center as in X^Tm^ control (Fig. 1C). This aberrant structure was made of a mixture of mineralized cartilage (Col10^+^) and bone (Col1^+^) matrixes and was filled with XΔEX/Tm^+^ cells. It appeared that many XEX/Tm^+^ cells, especially toward the middle, were producing both Col10 and Col1 (Fig. 1C). Amounts of Col1 and Col10 by IF staining appeared in reverse correlation within the same cell (Fig. 1C). In addition, Col1 staining was lining the rod immediately adjacent to XΔEX/Tm^+^ bone cells (Fig. 1C). In the p21 X/ΔEX^Tomato^ femur, Saf-O staining and anti-Col10 IF revealed an increased trabecular volume and a broadened hypertrophic zone of the distal growth plate (Fig. 1C). Essentially all XΔEX/Tm^+^ cells at the metaphyseal region were in direct contact with either the trabeculae or the cortices, and very few were dispersed in the bone marrow (Fig. 1C). The proximal marrow cavity was occupied by a rod-shaped mineralized tissue comprised of XΔEX/Tm^+^ cells similar to that of p2, suggesting that the anomalous structures might be remnants from earlier stages (Fig. 1C). By 1 month of age, the distal phenotype became more pronounced (Supplementary Fig. S3c), whereas the proximal aberrant structure gradually ceased to exist.

Collectively, these data indicated an inverse correlation between the number of non-trabecula-bound Tm^+^ cells and trabecular volumes in chondrocyte-conditional β-catenin mutants. We speculated that stromal non-trabecula-bound reporter^+^ cells may be precursors to trabecula-bound reporter^+^ cells.

### β-Catenin discretely regulates chondrocytes to mesenchymal progenitor cells (C-MPCs) and subsequent C-MPCs to mature osteoblast processes

**Fig. 2:**
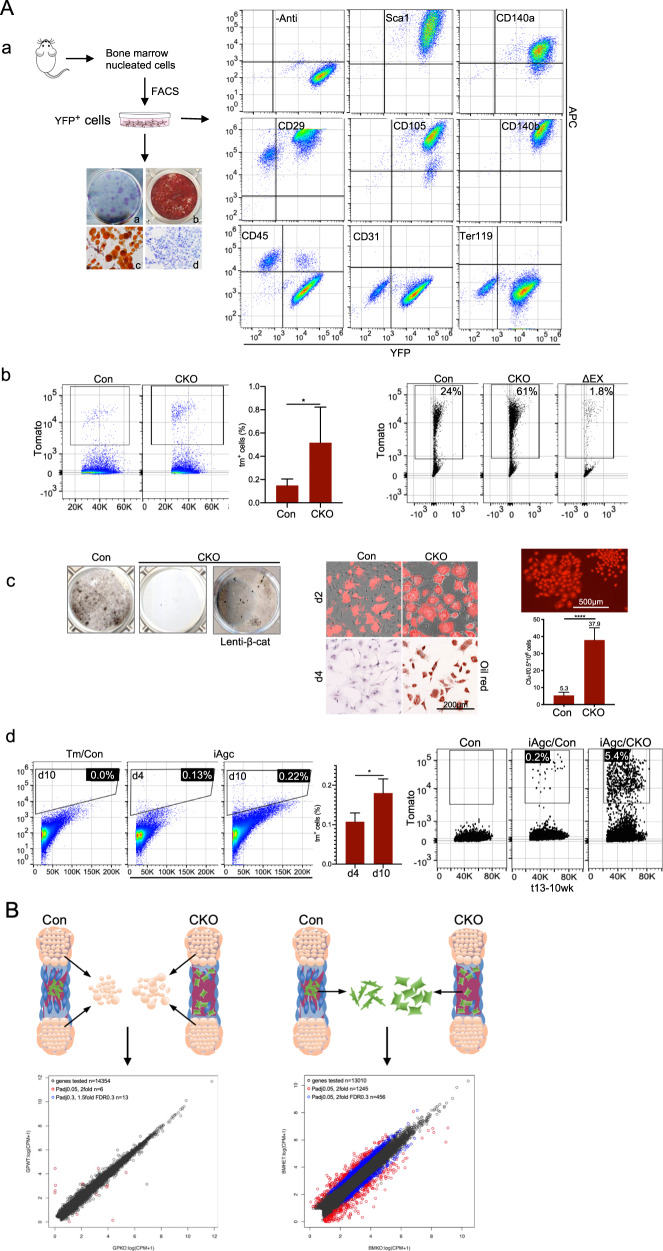
Characterization and expression profiling of C-MPCs. **A**:. **a** YFP^+^ C-MPCs were sorted from fresh stromal cells of X^YFP^ mice. After being propagated in culture, YFP^+^ C-MPCs were evaluated for MSC characteristics and marker expressions. Left top: marrow cell isolation scheme. Left bottom: **a** CFU-; **b** Alizarin red; **c** Oil red; **d** Alcian blue staining of YFP^+^ C-MPCs. Right panel: FACS analyses of MSC, hematopoietic, and endothelial cell marker expression of the YFP^+^ stromal cells sorted from 2-month-old X^YFP^ mice. **b**. Quantitative comparison of C-MPCs from X/CKO^Tomato^ (CKO), X/ΔEX^Tomato^ (ΔEX) mutant and X^Tomato^ (Con) control mice. Left: bone marrow cells were isolated from 5-week-old mice and directly subjected to FACS, *n* = 6, *p < 0.05. Right: bone marrow cells isolated from 3-week-old mice were cultured and then subjected to FACS. **c**. Tm^+^ cells were sorted from stromal cells and subjected to differentiation assays in vitro. Left: Von Kossa staining of Tm^+^ stromal cells after being cultured in osteogenic media for 21 days. Lenti-β-catenin-infected β-catenin^–^ Tm^+^ stromal cells from X/CKO^Tomato^ mice were able to form mineralized nodules. Middle: in vitro adipogenic assay: after being induced for 2 days, the β-catenin^–^ Tm^+^ stromal cells already became adipocytes shown by Oil red staining, not the control C-MPCs. Right: CFU-F image of the β-catenin^–^ Tm^+^ stromal cells and quantitative comparison of Tm^+^ CFU-Fs, *n* = 6, ****p < 0.0001. **d**. Left: p10 *ROSA26R-Tomato* (Tm/Con) and *Agc1-CreERT2;ROSA26R-Tomato* (iAgc) mice were injected with tamoxifen and bone marrow cells were analyzed by flow at day 4 and day 10 post treatment, *n* = 3, *p < 0.05. Right: p13 *Ctnnb1*^*fl/+*^*;ROSA26R-Tomato* (Con), *Agc1-CreERT2;Ctnnb1*^*fl/+*^*;ROSA26R-Tomato* (iAgc/Con) and *Agc1-CreERT2;Ctnnb1*^*fl/fl*^*. ROSA26R-Tomato* (iAgc/CKO) mice were injected with tamoxifen. After a 10-week chase, bone marrow cells were harvest for flow analysis. **B** RNA-seq analyses. Illustration of sample preparation and gene expression log-log plot. See legend in (**A**) for mice detail. C-MPC RNAs were isolated from C-MPCs sorted from fresh stromal cells of 5-week-old mice. Chondrocyte RNAs were extracted from chondrocytes dissected from growth plates of p6 mice.

To establish the identity of non-trabecula-bound stromal reporter^+^ cells, the total marrow stromal cells were collected from *Col10a1-Cre*;*ROSA26R-YFP* (X^YFP^) mice and sorted for YFP-positive (YFP^+^: 0.08%/2.5-month, 0.16%/5.5-month) cells by fluorescence-activated cell sorting (FACS) (Supplementary Fig. S4a). The sorted YFP^+^ cells were grown in culture and then harvested for analysis. Almost all YFP^+^ stromal cells showed positive signals for MSPC markers: Sca1^+^(99.81%), CD140a^+^(90.85%), CD140b^+^(99.97%), CD105^+^(96.78%), and negative signals for hematopoietic cell marker CD45, endothelial cell marker CD31, and erythroid cell marker Ter119 (Fig. 2Aa). Alternatively, we analyzed MSPC marker expression of fresh prepared stromal cells from X^Tomato^ mice and found that fractions of the Tm^+^ stromal cells showed positive signal for CD140a (11.9%) and CD105 (10.9%) (Supplementary Fig. S4b). Moreover, the YFP^+^ stromal cells were clonogenic and exhibited mesenchymal tri-lineage capacities in vitro (Fig. 2Aa).

*Osx* is expressed in bone marrow mesenchymal progenitor cells (MPCs)^23^. To evaluate *Osx* expression in chondrocyte-derived non-trabecula-bound stromal cells, we did experiments using *Col10a1-Cre*;*Osx*^*fl/+*^ (X/Osx^fl/+^) mice, in which *Osx*-expressing cells are identified by GFP upon Cre-mediated LoxP recombination^24^. Taking the approach shown in Supplementary Fig. S4, we found that approximately 35% of the adherent stromal cells were GFP^+^ cells (Supplementary Fig. S4c).

To confirm the histological quantification of non-trabecula-bound reporter^+^ cells shown in Fig. 1Ab, marrow stromal cells were collected from 5- to 6-week-old X^Tomato^ (Con) and X/CKO^Tomato^ (CKO) mice for flow analysis. It revealed an elevated percentage of Tm^+^ stromal cells from CKO mice compared to that from control mice (CKO/0.52% vs Con/0.15%, *n* = 6, *p < 0.05) (Fig 2Ab). Alternatively, marrow stromal cells of 3-week-old X^Tomato^ (Con), X/CKO^Tomato^ (CKO), and X/ΔEX^Tomato^ (ΔEX) mice were collected and grown in culture. The attached cells were analyzed by FACS. As shown in the fresh isolated marrow cells (Fig. 2Ab), there was an increase in the percentage of β-catenin-Tm+ stromal cells (61.2%) and deep drop in percentage of Tm+ stromal cells (1.8%) compared to that of control Tm+ stromal cells (24.2%) (Fig. 2Ab) from X/ΔEXTomato mice. These results validated histological quantification (Fig. 1Ab). We isolated the Tm+ stromal cells from the X^Tomato^ mice using the same approach as in Fig. 2Aa and found that they were negative for CD45 and CD31 proteins (Supplementary Fig. S4d).

Unlike the chondrocyte-derived Tm^+^ stromal cells from control mice (Fig. 2Aa, Ac), the ß-catenin^–^Tm^+^ stromal cells completely failed to form any mineralized nodules while intensely favoring adipogenic differentiation (Fig. 2Ac and Supplementary Fig. S6). In addition, both control Tm^+^ and ß-catenin^–^Tm^+^ cells were capable of forming CFU-Fs. We observed a robust increase in the number of CFU-Fs from X/CKO^Tomato^ stromal cells compared to those from X^Tomato^ mice (Fig. 2Ac). Furthermore, after being transduced by lenti-β-catenin, the non-trabecula-bound ß-catenin^–^Tm^+^ stromal cells reinstated osteoblastic differentiation (Fig. 2Ac), implying that the cell-autonomous ß-catenin deficiency was possibly accountable for the altered differentiation potential.

Histological analyses of tamoxifen chase experiments in our previous report^1^ show that the non-trabecula-bound Tm^+^ stromal cells first appear in small numbers at the chondral-osteo junction of growth plate, and gradually increase in number and spread into marrow cavity. Here, we isolated stromal cells from tamoxifen-treated iAgc^Tomato^ (iAgc) and *ROSA26R-Tomato* (Tm/Con) mice after a 4- and a 10-day chase, and quantified Tm^+^ cell portions by FACS. Shown in Fig. 2Ad, the percentage of Tm^+^ stromal cells from the mice chased for 10 days was significantly higher than that for 4 days (0.18% vs 0.11%, *n* = 3, *p < 0.05). In a separate experiment, we administered tamoxifen to p13 *Ctnnb1*^*fl/+*^;*ROSA26R-Tomato* (Con), *Agc1-CreERT2*;*Ctnnb1*^*fl/+*^;*ROSA26R-Tomato* (iAgc/Con), and *Agc1-CreERT2*;*Ctnnb1*^*fl/fl*^;*ROSA26R-Tomato* (iAgc/CKO) mice, and after a 10-week chase, stromal cells were isolated and plated in culture. Flow analysis revealed a higher percentage of β-catenin^–^ Tm^+^ cells from iAgc/CKO animals compared to that from iAgc/Con mice (Fig. 2Ad), which was similar to that in X/CKO mice (Fig. 2Ad). These results validated findings shown in Fig. 1Ba, Bb. FACS analysis found the presence of iAgc/Tm^+^Sca1^+^ and iAgc/Tm^+^CD140a^+^ stromal cells in the marrow of iAgc^Tomato^ mice (Supplementary Fig. S5). Our data demonstrated that chondrocyte-derived non-trabecular-bound stromal cells were *Osx*-expressing cells and processed MSPC capacities. In addition, β-catenin^–^ Tm^+^ cells heavily favored adipogenic differentiation at the expense of osteogenic differentiation in a similar fashion to the *Osx*-Cre-mediated β-catenin^–^ MPCs^7^.

Together these results supported the idea that chondrocytes were able to give rise to a population of MPCs—C-MPCs, which is a subpopulation in the total *Osx*-expressing MSPC pool.

The finding of C-MPCs along with our previous observation of a sequential emergence of iAgc/Tm^+^ stromal cells followed by iAgc/Tm^+^GFP^+^ osteoblasts^1^ leads us to hypothesize that chondrocyte to osteoblast reprogramming may take place in at least two steps: chondrocytes to C-MPCs and subsequently C-MPCs to osteoblasts.

To delineate how precisely Wnt/β-catenin signaling governs chondrocyte to osteoblast transformation, we did two separate pairs of expression profiling comparisons: (1) between growth plate chondrocytes*;* and (2) between C-MPCs of X/CKO^Tomato^ mutant and control animals (Fig. 2B).

Total chondrocyte RNAs were extracted from the growth plate chondrocytes dissected from 7-day-old X/CKO^Tomato^ and control littermates, and total C-MPC RNAs were extracted from the non-trabecula-bound Tm^+^ stromal cells of 5-week-old X/CKO^Tomato^ and *Col10a1-Cre*;*Ctnnb1*^*fl/+*^;*ROSA26R-Tomato* (X/CHet^Tomato^) control mice. The RNA-seq expression profiling of growth plate chondrocytes showed only 23 genes with equal to or more than two-fold changes in expression levels, out of over ten thousand genes detected in the experiment (Table 1 and Supplementary Table S8a), consistent with anti-Col10 staining shown in Fig. 1Ac. In sharp contrast, the expression profile of ß-catenin^–^ C-MPCs was extensively different from that of control C-MPCs. A total of 1633 genes showed equal to or more than two-fold difference in expression levels, with 790 genes up and 843 genes downregulated (Table 1 and Supplementary Table S8b). This result is in line with the severely altered bone and marrow phenotype. The profiling results offered additional validity to our interpretation that ß-catenin activity in hypertrophic chondrocytes is not needed for their C-MPCs-forming activity. The ß-catenin^–^ C-MPCs expressed lower levels of osteoblast marker genes such as *Col1a1*, *Dmp1*, and *Bglap*, as one would expect (Table 2). However, expression levels of osteogenic transcription factors *Runx2*, *Osx*, and *Dlx5* were not found to be significantly changed (Table 2). The result was validated by qPCR (data not shown).

**Table 1:** Numbers of genes with equal or more than two-fold changes between β-catenin^–^ and control chondrocytes, and between β-catenin^–^ and control C-MPCs.

	≥2-fold	≤2-fold
Chondrocytes	9	14
C-MPCs	790	843

*p*adj < 0.001.

**Table 2:** Expression-level comparisons of selected osteoblastic genes between the β-catenin^–^ C-MPCs and control C-MPCs.

	Log2 fold	*p*adj
*Runx2*	0.719	0.028
*Osx*	1.028	0.0008
*Dlx5*	1.28	0.0005
*Alpl*	1.86	0.0002
*Col1a1*	−1.85	0.0003
*Dmp1*	−3.39	0.0002
*Bglap*	−1.99	0.0024

Collectively, the data indicate that loss of ß-catenin activity in hypertrophic chondrocytes did not prevent formation of C-MPCs, which nonetheless were dependent on ß-catenin function to differentiate into mature osteoblasts.

### β-Catenin negatively regulates p53 in MPCs including chondrocyte-derived progenitor cells (C-MPC)

**Fig. 3:**
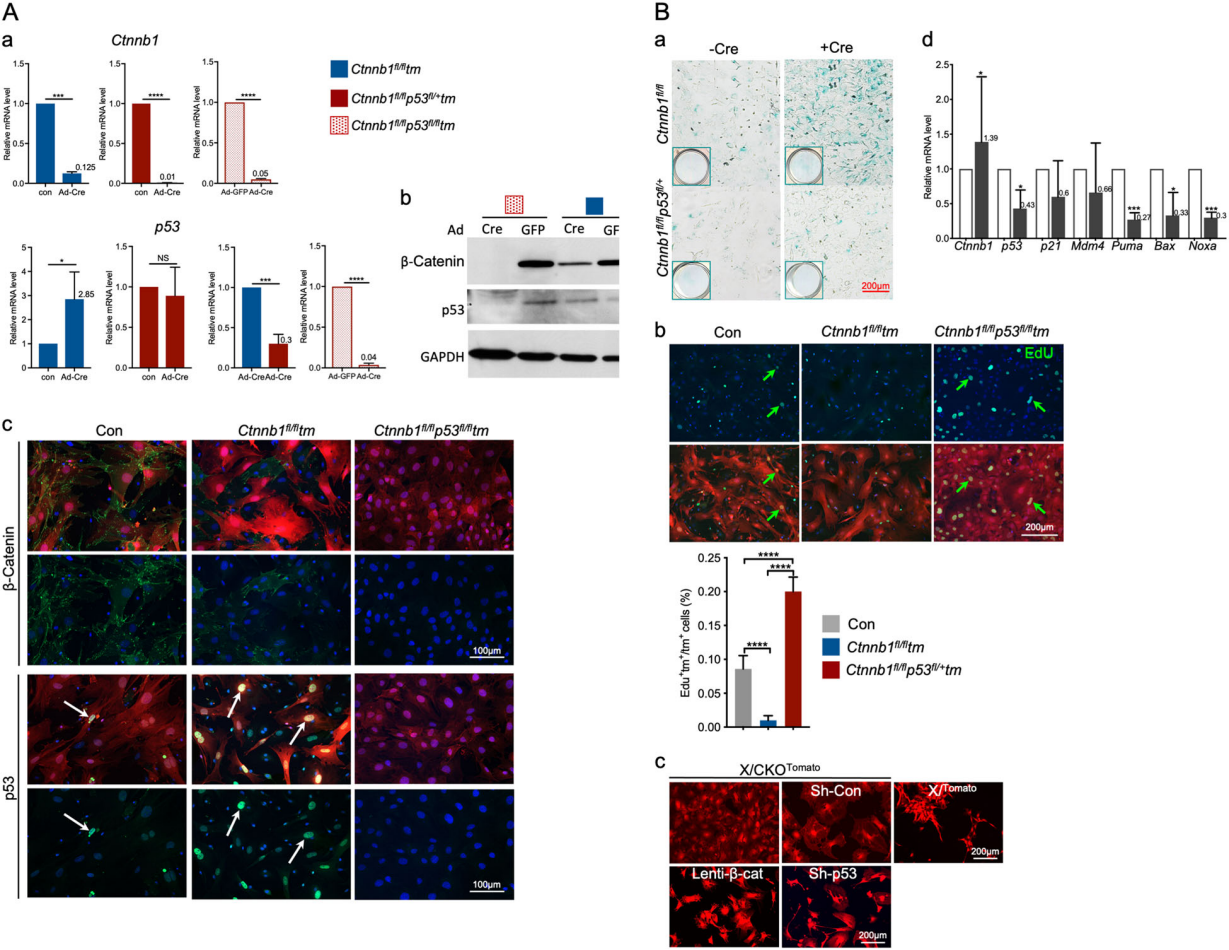
Elevated p53 activity in β-catenin^–^ MPCs. Marrow stromal cells were isolated from 6-week-old *Ctnnb1*^*fl/fl*^*ROSA26R-Tomato* (*Ctnnb1*^*fl/fl*^*tm*), *Ctnnb1*^*fl/fl*^*p53*^*fl/+*^*ROSA26R-Tomato* (*Ctnnb1*^*fl/fl*^*p53*^*fl/+*^*tm*) and *Ctnnb1*^*fl/fl*^*p53*^*fl/fl*^*ROSA26R-Tomato* (*Ctnnb1*^*fl/fl*^*p53*^*fl/fl*^*tm*) mice and were transduced by Ad-Cre or Ad-GFP (Con). **A** Determining p53 level in β-catenin^–^ MPCs. **a** RT-qPCR validation of *Ctnnb1* and *p53* expressions, normalized to *Rplp0*. Data represent the mean ± SEM, *p < 0.05, ***p < 0.001, ****p < 0.0001 by two-tailed *t*-test, *n* = 3. **b** Western blot analysis of MPCs. **c** Anti-β-catenin and anti-p53 ICC of Ad-Cre transduced MPCs. Con: ROSA26R-Tomato. White arrows indicate the nuclei with p53 staining. **B** p53 functional assays. **a** X-gal staining for senescent MPCs. **b** Proliferation assay. Green arrows indicate Edu^+^ MPCs. The graph shows the percentages of EdU^+^tm^+^ of total tm^+^ cells, ****p < 0.0001, *n* = 3. **c** Tm^+^ C-MPCs (β-catenin^–^ C-MPCs) were sorted from bone marrow of 5^-^week-old X/CKO^Tomato^ and X^Tomato^ mice. The β-catenin^–^ C-MPCs were transduced with Lenti-β-catenin, Lenti-shcon, Lenti-shp53. Images were taken with EVOS microscope. **d** qPCR of p53 target genes comparing the lenti-β-catenin transduced β-catenin^–^ C-MPCs (dark gray bars) to the lenti-con transduced β-catenin^–^ C-MPCs (open bars), shown in expression fold change, normalized to Rplp0, *p < 0.05, ***p < 0.001, *n* = 3.

Ingenuity pathway analysis (IPA) projected p53 as the top upstream regulator (Table 3) contributing to the cellular outcomes due to loss of ß-catenin. To validate, we did culture-based recombination experiments to achieve ß-catenin (*Ctnnb1*) and/or p53 deletions in MPCs. The qPCR confirmed that adeno-Cre (Ad-Cre) efficiently deleted *Ctnnb1* and/or *p53* conditional alleles (Fig. 3Aa). The Ad-Cre infected *Ctnnb1*^*fl/fl*^*tm* MPCs (ß-catenin^–^Tm^+^ MPCs) showed a 2.85-fold increase in *p53* expression compared to the mock treated *Ctnnb1*^*fl/fl*^*tm* MPCs (Fig. 3Aa). A slight increase of *p53* expression was detected in *Ctnnb1*^*fl/fl*^*tm* C-MPCs from X/CKO^Tomato^ mice relative to control C-MPCs (Supplementary Fig. S7). The p53 protein was slightly higher in the ß-catenin^–^Tm^+^ MPCs, in spite of incomplete ß-catenin deletion (Fig. 3Ab). Immunocytochemistry (ICC) validated that ß-catenin was efficiently removed from both ß-catenin^–^Tm^+^ and ß-catenin^–^p53^–^Tm^+^ MPCs, in reference to control MPCs (Fig. 3Ac). The nuclei of ß-catenin^–^Tm^+^ MPCs were intensely stained by anti-p53 antibody (green), while no signal was detected in control MPCs (Fig. 3Ac). The ß-catenin^–^Tm^+^ C-MPCs exhibited characteristic senescent cell morphology, being cube-shaped, much larger, and flatter with little or no dendritic extensions distinct from spindle-shaped control MPCs including C-MPCs. Acidic β-galactosidase (β-gal) assay revealed a higher number of β-gal^+^ cells in ß-catenin^–^ MSPC population. This phenotype was attenuated by removal of p53 (Fig. 3Ba). In addition, there was a significant decline in percentage of EdU^+^ ß-catenin^–^Tm MPSCs compared to that of control MPCs, and this reduction was entirely reversed by depleting p53 (Fig. 3Bb). Reintroducing β-catenin to β-catenin^–^Tm^+^ C-MPCs reversed their senescent cell-like morphology (Fig. 3Bc). A similar morphological transformation took place in β-catenin^–^Tm^+^ C-MPCs infected by lenti-shp53 (Fig. 3Bc). Furthermore, lenti-β-catenin-infected ß-catenin^–^Tm C-MPCs lowered expressions of p53 targets, including *p21*, *Mdm4*, *Puma*, *Bax*, and *Noxa* (Fig. 3Bd).

**Table 3:** List of the top 3 transcription factors projected by IPA Upstream Regulator analysis.

Upstream regulator	Expr log ratio	Predicted state	Activation *z*-score	*p* value of overlap	Target molecules in database
TP53		Activated	2.693	8.06E–30	342
MYC	−0.561	Inhibited	−2.108	3.54E–09	138
TRIM24		Activated	3.066	0.00000366	33

Collectively, these data substantiated that p53 activity is indeed elevated in ß-catenin^–^ MPCs including C-MPC subpopulation, indicating that Wnt/ß-catenin negatively regulates p53 in these cells.

### Deleting p53 from β-catenin^–^ MPCs fully restored osteogenic differentiation

**Fig. 4:**
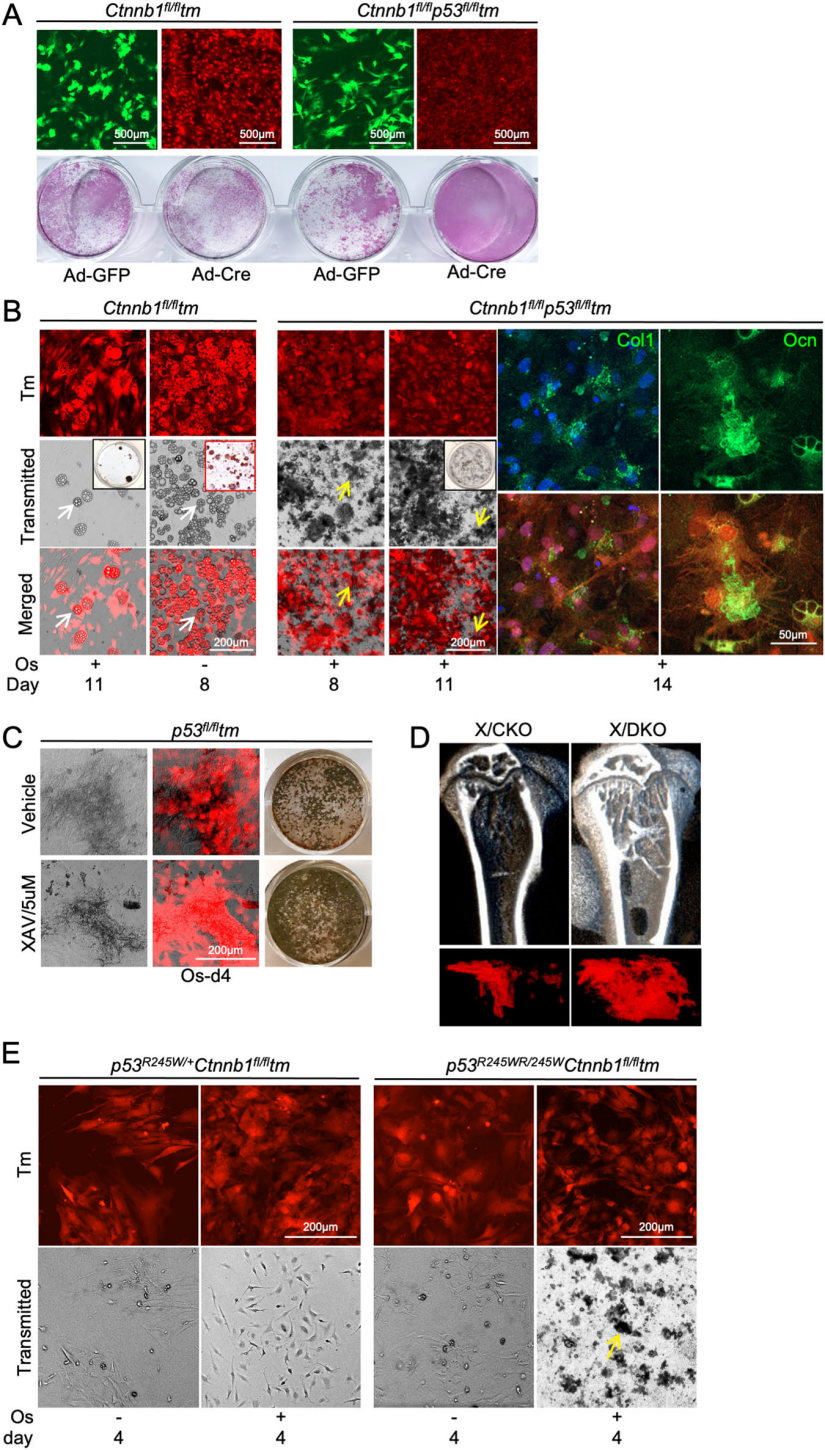
Evaluating p53 role in context of β-catenin osteogenic activity in MPCs. Mutant and control MPCs in this figure are same as in Fig. 3. **A** β-Catenin^–^p53^–^ MPCs displayed higher alkaline phosphatase activity than that of β-catenin^–^ MPCs. **B** Ad-Cre transduced MPCs were induced for osteogenic differentiation in vitro and assayed for mineralization by Von Kossa (black box) and for adipocytes by Oil Red (red box). Cells were imaged live using EVOS microscope. White arrows: adipocytes; yellow arrows: mineralized nodules. ICC with anti-Col1 and anti-Ocn antibodies validated ossification of β-catenin^–^p53^–^ MPCs. **C** p53^–^ MPCs were treated with either DMSO (vehicle) or 5 µM XAV939 and induced for osteoblastic differentiation. It only took as few as 4 days for the XAV939-treated p53^–^ MPCs to mineralize, shown by Von Kossa. **D** The μCT images of humeri from 6-week-old X/CKO and X/DKO mice, metaphyseal trabeculae excluding cortices shown in pseudo-red. **E** MPCs isolated from the *p53*^*R245W/+*^*;Ctnnb1*^*fl/fl*^*;ROSA26R-Tomato* and *p53*^*R245W/R245W*^*;Ctnnb1*^*fl/fl*^*;ROSA26R-Tomato* mice were transduced by Ad-Cre. The β-catenin^–^p53^R245W/R245W^tm MPCs formed mineralized nodules 4 days after osteo-induction. Yellow arrow indicates mineralized bone nodule.

The ß-catenin^–^p53^–^Tm^+^ MPCs showed higher ALP activity than the ß-catenin^–^Tm^+^ MPCs (Fig. 4A). Consistently, the ß-catenin^–^p53^–^Tm^+^ MPCs fully mineralized in vitro shown by Von Kossa staining and by Col1 and Ocn productions revealed by ICC (Fig. 4B), demonstrating that p53 depletion from β-catenin^–^ MPCs sufficiently released osteogenic inhibition. To verify, we used an alternative method to attain β-catenin inactivation. Shown in Fig. 4C, β-catenin inhibitor XAV939-treated p53^–^Tm^+^ MPCs maintained osteogenic capacity as the vehicle-treated p53^–^Tm^+^ MPCs. The uCT imaging revealed that trabecular bone loss in the X/CKO mice was mostly recovered in the Col*10a1-Cre*;*Ctnnb1*^*fl/fl*^;*p53*^*fl/fl*^ (X/DKO) mice (Fig. 4D), presenting in vivo proof for the essential negative role of p53 in ß-catenin^–^ MSPC osteogenic defect. To evaluate if p53 DNA-binding activity is involved in its anti-osteogenic function, we crossed *p53*^*R245W*^ allele to *Ctnnb1*^*fl/fl*^*tm* mice to generate *p53*^*R245W/+*^*Ctnnb1*^*fl/fl*^*tm* and *p53*^*R245W/R245W*^*Ctnnb1*^*fl/fl*^*tm* mice. The p*53*^*R245W*^ allele contains a hot spot mutation in DNA-binding domain that completely abolishes its DNA-binding ability^25^. It took as few as 4 days for ß-catenin^–^p53^R245WR245W^Tm^+^ MPCs to mineralize in in vitro osteogenic differentiation assay (Fig. 4E), implying that p53’s anti-osteogenic ability is dependent on its DNA-binding activity.

**Fig. 5:**
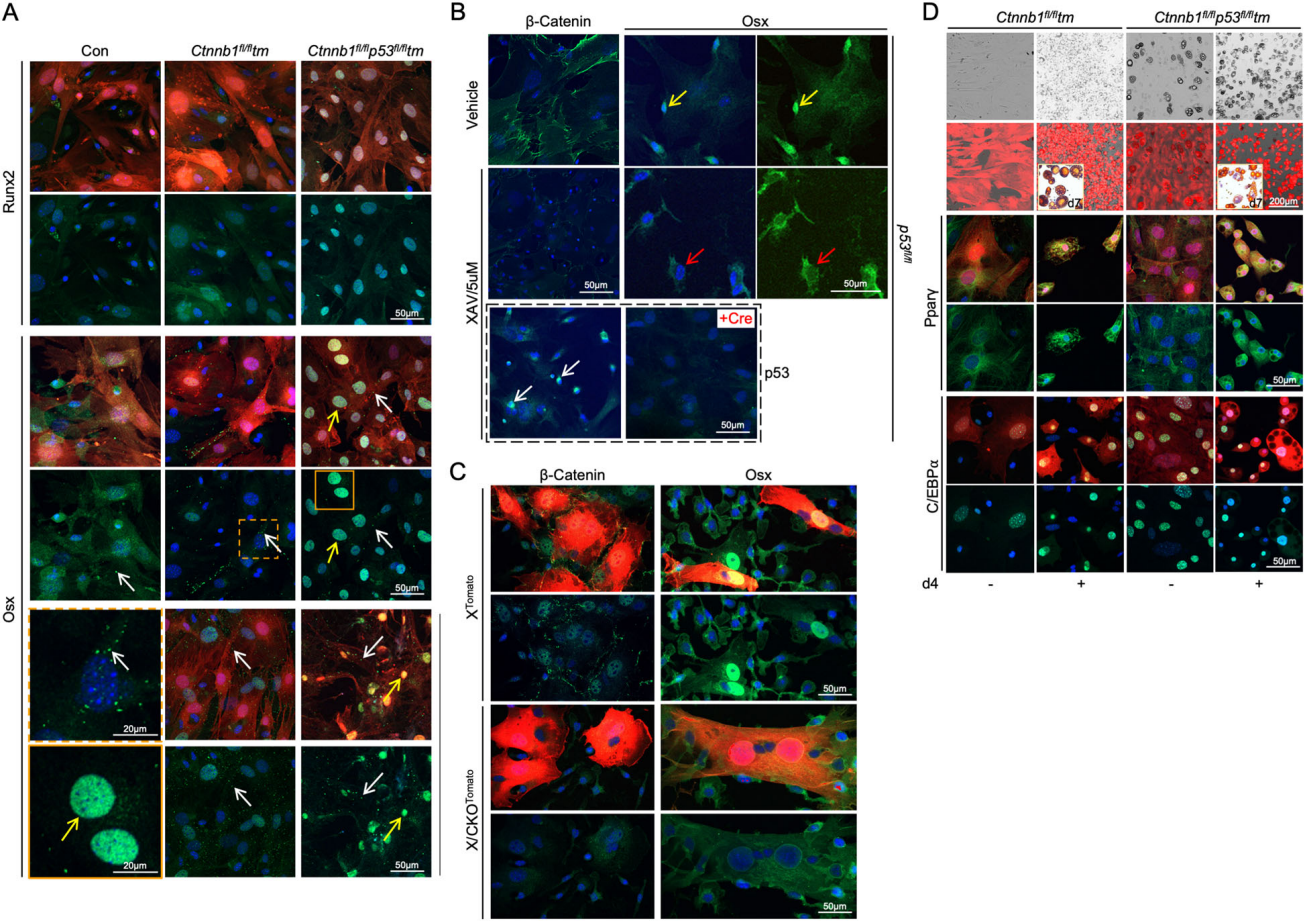
Elevated p53 in the nuclei of β-catenin^–^ MPCs. **A** Confocal images of anti-Runx2 and anti-Osx ICC of Ad-Cre-treated MPCs. Con: *ROSA26R-Tomato*. The images with dotted and solid orange lines at bottom left show magnified areas indicated by corresponding orange squares. Yellow arrows: Osx^+^ nuclei. White arrows: Osx^+^ granules. **B** MPCs were isolated from *p53*^*fl/fl*^ mice and induced for osteoblastic differentiation for 7 days in the presence of DMSO (vehicle) or XAV939. ICC revealed evident β-catenin and Osx (yellow arrows) signal on vehicle-treated MPCs, whereas XAV939-treated MPCs showed no β-catenin, strong p53 nuclear staining (white arrows), and Osx signal outside of nuclei (red arrows). Only MPCs of the image labeled “+Cre” were infected with Ad-Cre. **C** Anti**-**β-catenin and anti-Osx staining of control and β-catenin^–^ C-MPCs isolated from 3-week-old X^Tomato^ and X/CKO^Tomato^ mice. **D** Isolation of MPCs is detailed in Fig. 3A. The Ad-Cre-treated MPCs were cultured with or without adipogenic stimuli. Adipogenic differentiation was evaluated at day 4 by anti-Pparγ and anti-C/EBPα ICC and at d7 by Oil Red staining shown in the images with yellow outlines. Top two rows of images are taken using EVOS microscope from live cells.

ICC revealed that in control and β-catenin^–^Tm^+^ MPCs, Runx2 was barely detectable in the absence of osteogenic stimuli except for sporadic relatively brighter Runx2^+^ granules in the cytoplasm of control MPCs (Fig. 5A). In comparison, the nuclei of ß-catenin^–^p53^–^Tm^+^ MPCs showed relatively more apparent Runx2 signal (Fig. 5A). Meanwhile, Osx was detected in both cytoplasmic and nuclear compartments of control MPCs, with a slightly brighter nuclear peripheral staining and a few in granule-form (Fig. 5A), whereas in the β-catenin^–^Tm^+^ MPCs, Osx was devoid from the nuclei and was only observed in granule-from in cytoplasm (Fig. 5A). Remarkably, the nuclei of β-catenin^–^p53^–^ Tm^+^ MPCs were intensely stained by anti-Osx antibody and the signal became even stronger after 7 days of osteogenic induction, while in the β-catenin^–^Tm^+^ MPCs, osteogenic induction led to an increase in the number of Osx^+^ granules in cytoplasm, verified by apparent nuclear staining on the neighboring Tm^–^ control cells (Fig. 5A). To validate, we did ICC on DMSO and XAX939-treated MPCs (Figs. 4C and 5B). The anti-β-catenin and anti-p53 ICC confirmed sufficient inhibition of β-catenin and upregulation of p53 in the XAX939-treated MPCs (Fig. 5B). Similar to β-catenin^–^Tm^+^ MPCs shown in Fig. 5A, Osx was mostly localized outside the nuclei of XAX939-treated MPCs, in contrast to vehicle-treated MPCs (Fig. 5B). In addition, the β-catenin^–^Tm^+^ C-MPCs from X/CKO^Tomato^ mice exhibited Osx cellular localization pattern (Fig. 5C) similar to the β-catenin^–^Tm^+^ (Fig. 5A) and the XAX939-treated MPCs (Fig. 5B).

β-Catenin^–^ C-MPCs were more adipogenic than their control counterparts (Fig. 2Ac and Supplementary Fig. S6), consistent with β-catenin’s pro-osteo/anti-adipogenic property. We queried whether p53 had any role in β-catenin’s anti-adipogenic function. We found that the β-catenin^–^p53^–^Tm^+^ MPCs maintained as much enhanced adipogenic capacity as the β-catenin^–^Tm^+^ MPCs (Fig. 5D), meaning that removal of p53 from β-catenin^–^Tm^+^ MPCs had no impact on β-catenin deficiency-mediated adipogenic acceleration. In accordance, ICC with anti-C/EBPa and anti-Pparr antibodies showed no discernable differences between the β-catenin^–^p53^–^Tm^+^ and β-catenin^–^Tm^+^ MPCs (Fig. 5D). We noticed that an ample number of β-catenin^–^p53^–^ MPCs were adipocytes in their default state (Fig. 5D).

## Discussion

Despite emerging recognition of p53’s roles in non-transformed cells, the level of understanding of its anti-osteogenic function has barely progressed since over a decade ago. Given that p53 is the most frequently mutated gene in osteosarcoma (OS)^26^, which arises from osteoblastic lineage cells, defining its role in osteoblast differentiation holds special value to the hunt for etiological and pathogenetic mechanisms underlying OS tumorigenesis and an ultimate cure for OS.

The revelation of chondrocytes as an innate source of osteoblasts has received a divided response, largely due to the lack of a plausible explanation. Little has been accomplished since its discovery more than 6 years ago. Two independent studies found that there are reduced numbers of Chon-obs in mice with Wnt/ß-catenin deletion in chondrocytes^1,2^. Both groups tested a variety of hypothetical causes for the declines including potential alteration in proliferation and/or apoptosis in chondrocytes and/or Chon-obs. Their findings are conflicting and are inadequate to justify the Chon-obs phenotype. In our study we did not find any evidence for the chondrocyte-derived “Osx” cells^1^ or for the severe defects in proliferation in both ß-catenin^–^ chondrocytes and Chon-obs^2^ (Supplementary Fig. S8a, b).

The sequential emergence phenomenon of chondrocyte-derived non-trabecula-bound stromal cells and Chon-obs^3^ along with the “de-differentiation” feature shared by various organisms undergoing trans-differentiation^27^ suggests that chondrocytes to osteoblasts reprogramming may also follow a similar “de-differentiation” mechanism.

Here we gathered several lines of evidence in favor of our hypothesis: (1) the C-MPCs’ progenitor cell-like properties (Fig. 2Aa, Ac and Supplementary Fig. S4). Although we do not yet fully understand their precise identity, our data are sufficient to distinguish them from differentiated cells such as mature chondrocytes and osteoblasts. The marker profiles of Tm^+^ C-MPCs in fresh prepared samples represented a snapshot of these cells in various progenitor states and should be expected to differ from that of culture-synchronized C-MPCs (Fig. 2Aa). (2) Sequential temporal relationship of Cre-induced reporter^+^ cells in the order of: chondrocytes (Tm^+^) – C-MPCs (Tm^+^GFP^+^ cells) – Chon-obs (Tm^+^GFP^+^) shown by tamoxifen chase experiments. (3) The finding of discrete regulations by Wnt/ß-catenin substantiated by RNA-seq profiling and histological and differentiation analyses favors the stepwise idea (Fig. 2B and Supplementary Fig. S8).

The full rescue by solely deleting p53 from ß-catenin^–^ MPCs suggested that ß-catenin likely does not directly regulate *Runx2* and *Osx* promoter activity. The partial rescue of trabecular volume in X/DKO mice solidified the key negative role of p53 in the context of Wnt/ß-catenin pro-osteogenic function.

Evaluation of whether there is any change in osteoclast differentiation compared to the X/CKO mice would prove to be an interesting follow-up.

Since p53 and Osx were not concurrently localized in the same cellular compartment of ß-catenin^–^ MPCs, it is implausible that a p53-Osx physical interaction could be the reason for osteogenic inhibition^28^. Of great ongoing interest is further understanding of the granule-form of Osx and its transport regulation. Likewise, p53 upstream events triggered by ß-catenin signaling are equally important and yet to be elucidated.

**Fig. 6:**
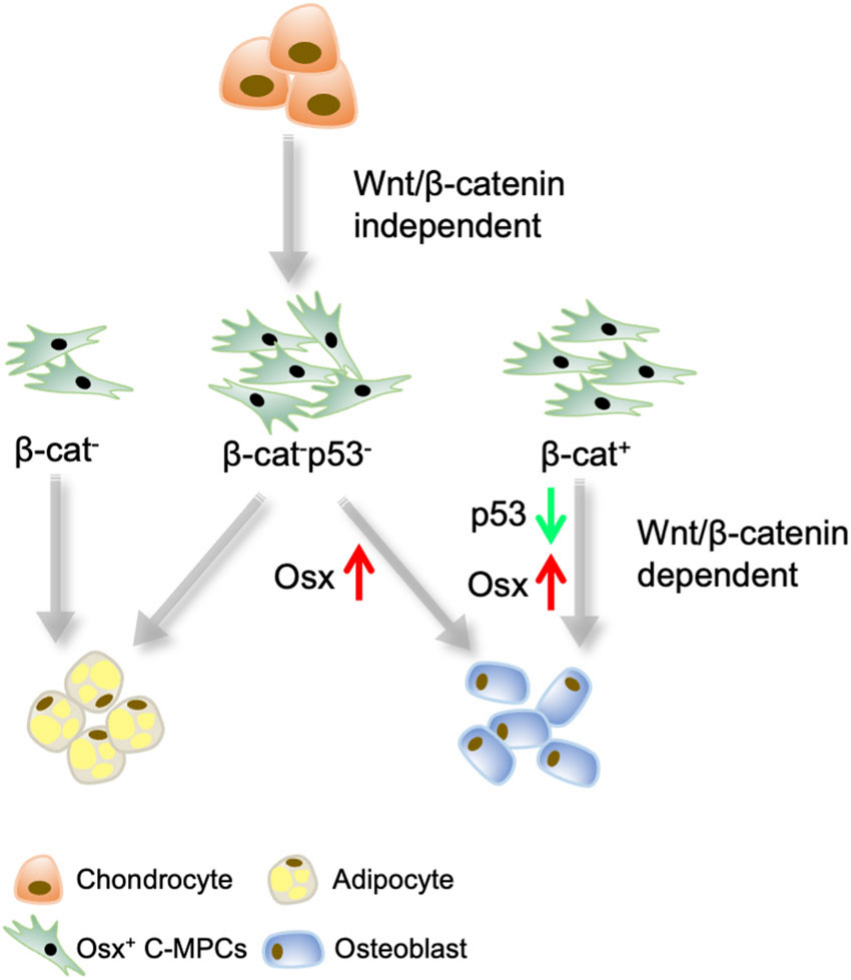
Illustration of Wnt/ß-catenin regulatory mechanism in chondrocyte to osteoblast process. Chondrocyte to osteoblast reprogramming proceeds in at least two steps: Wnt/ß-catenin-independent Step 1: chondrocytes give rise to a population of mesenchymal progenitor cells (C-MPCs), a subset of MPCs and Wnt/ß-catenin-dependent Step 2: Wnt/ß-catenin suppresses p53 to grant C-MPCs osteoblastic differentiation.

Our study advanced understanding in two fundamental subjects: (1) identification of p53 as a key node negatively involved in Wnt/ß-catenin-mediated osteogenesis; (2) revealing of a stepwise chondrocyte to osteoblast process independently regulated by Wnt/ß-catenin signaling (Fig. 6). Given the broad roles of both p53 and canonical Wnt signaling, we hope that our basic findings will translate to benefit clinical research beyond the scope of bone disease.

## Materials and methods

### Experimental animals

*Col10a1-Cre*^29^, *Agc1-CreERT2*^30^, *Osx*^*fl/fl*31^, *Ctnnb1*^*fl/fl*^, *Ctnnb1EX3*^*fl/f*^, *p53*^*R245W*^^25^, *p53*^*fl/fl*^, and *2.3Col1a1-GFP* mice have been described. *Ctnnb1EX3*^*fl/fl*^ mice were provided by Dr Makoto Taketo of Kyoto University. *2.3Col1a1-GFP* mice were provided by Dr David Rowe of University of Connecticut. *Ctnnb1*^*fl/fl*^ (*B6.129-Ctnnb1*^*tm2Kem*^*/KnwJ*, Stock No: 004152), *ROSA26R-tdTomato* (also as Ai9) (Gt (ROSA)26Sortm9(CAG-tdTomato) Hze, Stock No: 007909) and *ROSA26R-YFP* (B6.129 × 1-*Gt (ROSA)26Sor*^*tm1(EYFP)Cos*^/J, Stock No: 006148) mice were purchased from the Jackson Laboratory. Tamoxifen (Sigma-Aldrich T-5648) was injected intraperitoneally at 1.5–3.0 mg/10 g body. All animals were housed in pathogen-free conditions, procedures followed the rules and regulations of AAALAC and were approved by Institutional Animal Care and Use Committee of University of Texas MD Anderson Cancer Center.

### Isolation of bone marrow stromal cells

Bone marrow nonhematopoietic stromal cells were isolated as described^32^. Total bone marrow cells were cultured in alpha-MEM media containing 20% FBS under 5% O_2_ up to around 10 days. Attached cells were passaged for expansion.

### Cell sorting and flow cytometry

Cell sorting experiments were performed on Arial II Cell Sorter (BD Bioscience). Flow cytometry analyses were carried out using Gallios 561 (Beckman Coulter). Services were provided by MDACC NORTH Campus Flow Cytometry and Cellular Imaging Core Facility. Data were analyzed using FlowJo or Kaluza.

### CFU-F and in vitro differentiation assays

Bone marrow plugs flushed out of femurs, tibias, and humeri were treated with Collagenase I (3 mg/ml, Worthington) and Dispase II (4 mg/ml, Roche) as previously described^32^. Collected cells were plated 0.5–1 × 10^6^/well in 6-well plates and cultured in alpha-MEM media/20% FBS under 5% O_2_ for 10 days without changing media and were stained with crystal violet with methanol. R&D Mouse Mesenchymal Stem Cell Functional Identification Kit (R&D) was used for in vitro osteogenic, adipogenic, and chondrogenic assays.

### Immunofluorescence staining

Long bones were fixed in freshly made 4% paraformaldehyde/PBS (pH 7.2) at 4 °C overnight and changed to 14% EDTA for 2–7 days at 4 °C. Decalcified bones were immersed in 30% sucrose/PBS for 1 h before embedded in OCT compound. Then, 8–12 µm sections were prepared using CryoStar NX70 Cryostat. Hyaluronidase treatment (2 mg/ml in PBS [pH 5.0]) was used for antigen retrieval, 20’ for embryonic or 30’ for postnatal tissue at 37 °C. Primary antibodies used were anti-mouse collagen type I (Millipore AB765P, 1:50) and anti-Collagen X antibody (ab58632, 1:200). Secondary antibodies were Alexa fluor 488 goat anti-rabbit IgG and Alexa fluor 488 goat anti-mouse IgG (Molecular probes). IF sections were mounted with Prolong Gold antifade reagent with DAPI (Invitrogen P36931).

### Confocal microscopy imaging

Fluorescence images were captured using A1 Laser scanning confocal microscope by Nikon Instruments at Microscopy Laboratory in the Department of Genetics at MDACC.

### RNA-seq and analysis

Tm^+^ C-MPCs were sorted by FACS from fresh marrow of 5-week-old X/CKO^Tomato^ and X/CHet^Tomato^ mice, two of each genotype. Total RNAs were isolated using Quick-RNA Micro-prep kit (Zymo research), followed by additional DNase treatment and purification (RNA clean and concentrator-5 kit, Zymo research). Around 100 ng total RNAs of each sample was sent to Sequencing and Microarray Facility at MDACC for strand-specific RNA-Seq analysis. Libraries were made with Illumina’s TruSeq Stranded Total RNA Library Prep Kit and were sequenced in 76 paired-end format on Illumina Next Generation Sequencing-HiSeq4000.

### X-gal staining

X-gal staining procedure was as described^33^.

### Lentivirus and adenovirus transduction

Ad5-cmv-GFP and Ad5-cmv-Cre were purchased from Baylor College of Medicine’s Vector Development Lab. Primary stromal cells were transduced with Ad5-cmv-Cre or Ad5-cmv-GFP at a concentration of 5000 pv/cell (8 µg/ml polybrene). After around 24 h, fresh media was added to replace media containing adenoviruses. Lenti-ß-catenin was generously provided by Andrew Gladden of the Genetics department. Lentivirus plasmids pGIPZ2 (empty vector), pGIPZ3 (non-specific shRNA), and pGIPZ-shp53 were purchased from MDACC Functional Genomics Core.

### Real-time qPCR

Quick-RNA Micro-prep kit (Zymo Research) was used to extract total RNAs. cDNAs were synthesized using amfiRivert cDNA Synthesis Platinum Master Mix (Gendepot). qPCR reactions were made using amfiSure qGreen Q-PCR Master Mix(2X), Low Rox (Gendepot), and QuantStudio 6 (Applied Biosystems). Primer sequences were designed using Integrated DNA Technologies’s PrimerQuest tool.

### Statistics

Statistical analysis was calculated by two-tailed, unpaired Student’s *t*-test in GraphPad Prism 7.0. The mean values were presented. The error bars indicated SEM.

## Supplementary information

Supplementary figure legends

Supplementary Figure S1

Supplementary Figure S2

Supplementary Figure S3

Supplementary Figure S4

Supplementary Figure S5

Supplementary Figure S6

Supplementary Figure S7

Supplementary Figure S8

Supplementary Figure S9

## Data Availability

The data reported in the current study are available from the corresponding authors upon request.
