# Supplementary material for: Wnt/ß-catenin-mediated p53 suppression is indispensable for osteogenesis of mesenchymal progenitor cells
Source: Cell Death Dis. 2021 May 21;12(6):521. doi: 10.1038/s41419-021-03758-w (PMC8139956; doi:10.1038/s41419-021-03758-w)
Supplement: Supplementary file 1 — Supplementary figure legends [file 41419_2021_3758_MOESM1_ESM.docx]

**Supplemental figure legends**

**Figure S1.** Confocal images of femurs without (left) and with anti-Col10 immunostaining, as well as Saf-O and H&E-stained femur images of 4-month (a) and 8-month-old (b) X^Tomato^ and X/CKO^Tomato^ mice. Yellow asterixis indicate growth plate remnants validated by anti-Col10 IF. White brackets (red in H&E): growth plates; White boxes denote magnified areas**.**

**Figure S2.** Confocal and H&E femur images of *Agc1-CreERT2;Ctnnb1^fl/+^;ROSA26R-Tomato* (iAgc/CHet^Tomato^) control and iAgc/CKO^Tomato^ mutant mice. p10 mice were injected with tamoxifen and chased for (a) 4 weeks, (b) 10 weeks and 10 months. White arrowheads indicate cortices. Brackets indicate growth plates. (c). Graphs show iAgc/Tm^+^ cell distribution, the cells were counted in three zones (z1, z2, z3) in distal to proximal order, z1 starts from chondral-osteo junction. con: iAgc/CHet^Tomato^; ko: iAgc/CKO^Tomato^.

**Figure S3.** Femurs of 2-month-old control and mutant mice were processed for (a) **μ**CT and (b) Toluidine blue (top) and TRAP (bottom) staining using plastic sections. (c). Confocal and Saf-O images of 1-month-old X/ΔEX^Tomato^ mice.

**Figure S4.** (a)**.** Total marrow nucleated cells were collected from 2.5- and 5.5-month-old X^YFP^ mice and sorted for YFP^+^ cells. (b)**.** Marker expression analyses of Tm^+^ cells from total marrow nucleated cells of 1-month-old X^Tomato^ mice. (c). Analyses using marrow cells of the *X/Osx^fl/+^* mice. Left panel: FACS analysis of GFP^+^ cell fraction in the total attached stromal cell culture from the 4-month-old *X/Osx^fl/+^* mice. (d). The marrow stromal cells from 2-month-old XTomato mice were cultured, followed by FACS to sort for the Tm^+^ cells. These Tm^+^ cells were further expanded in culture and then evaluated for CD45 and CD31 expressions.

**Figure S5.** (a). FACS analysis of the iAgc*/*Tm^+^ C-MPCs from tamoxifen treated iAgc^Tomato^ and *ROSAR26R-Tomato* (Con) mice, showing presence of Sca1^+^Tm^+^ and 140a^+^Tm^+^ C-MPCs from iAgc^Tomato^ mice, using APC conjugated anti-Sca1 and anti-CD140a antibodies. (b). FACS analysis of the iAgc^Tomato^ C-MPCs and Tm^+^GFP^+^ Chon-obs in the marrow and bone fractions. Cells were isolated from the Con, 2.3-GFP/Tomato and iAgc/2.3-GFP^Tomato^ mice, which were injected with tamoxifen at 2-week-old and chased for 4 and 7 weeks respectively.

**Figure S6.** A. qPCR analyses for adipocyte marker expression. The X/Tm^+^ C-MPCs were sorted from the bone marrow of 3-week X/CKO^Tomato^ and X^Tomato^ control mice, and induced for adipogenesis *in vitro*. Normalized to *Hprt*, n=3. Black bar: Con; Grey bar: X/CKO^Tomato^.

**Figure S7.** qPCR analyses using total RNAs isolated from C-MPCs of 2-month-old X/CKO^Tomato^ mutant and X/CHet^Tomato^ control mice.

**Figure S8.** RNA-seq heatmaps of the growth plate chondrocytes (a) and the C-MPCs (b).

**Figure S9.** (a). The bar graph of proliferating (EdU^+^) cells in the proliferating zones of growth plates. Left graph: p16 X^Tomato^ (con) and X/CKO^Tomato^ mice; Right graph: iAgc^Tomato^ (con) and iAgc/CKO^Tomato^ mice chased for 4 weeks post tamoxifen at p10. n=3. (b). The growth plates confocal images of *Ctnnb^fl/+^Tomato* (Con) and iAgc/CKO^Tomato^ mice. Line-circled areas define growth plates. White arrows indicate EdU^+^ cells.
