# Supplementary figures and images for: Wnt/ß-catenin-mediated p53 suppression is indispensable for osteogenesis of mesenchymal progenitor cells
Source: Cell Death Dis. 2021 May 21;12(6):521. doi: 10.1038/s41419-021-03758-w (PMC8139956; doi:10.1038/s41419-021-03758-w)

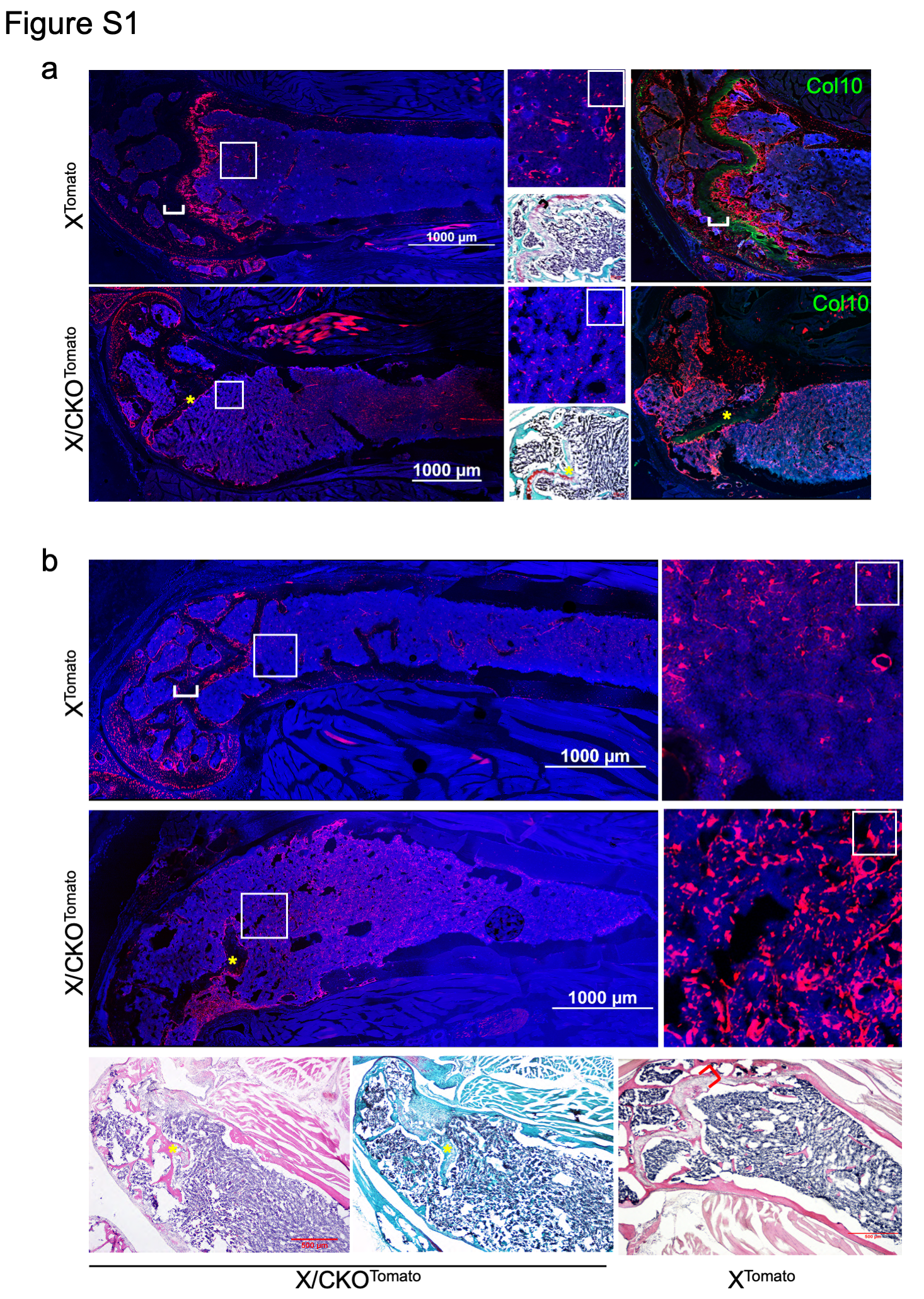

Supplement: Supplementary file 2 — Supplementary Figure S1 [file 41419_2021_3758_MOESM2_ESM.png]

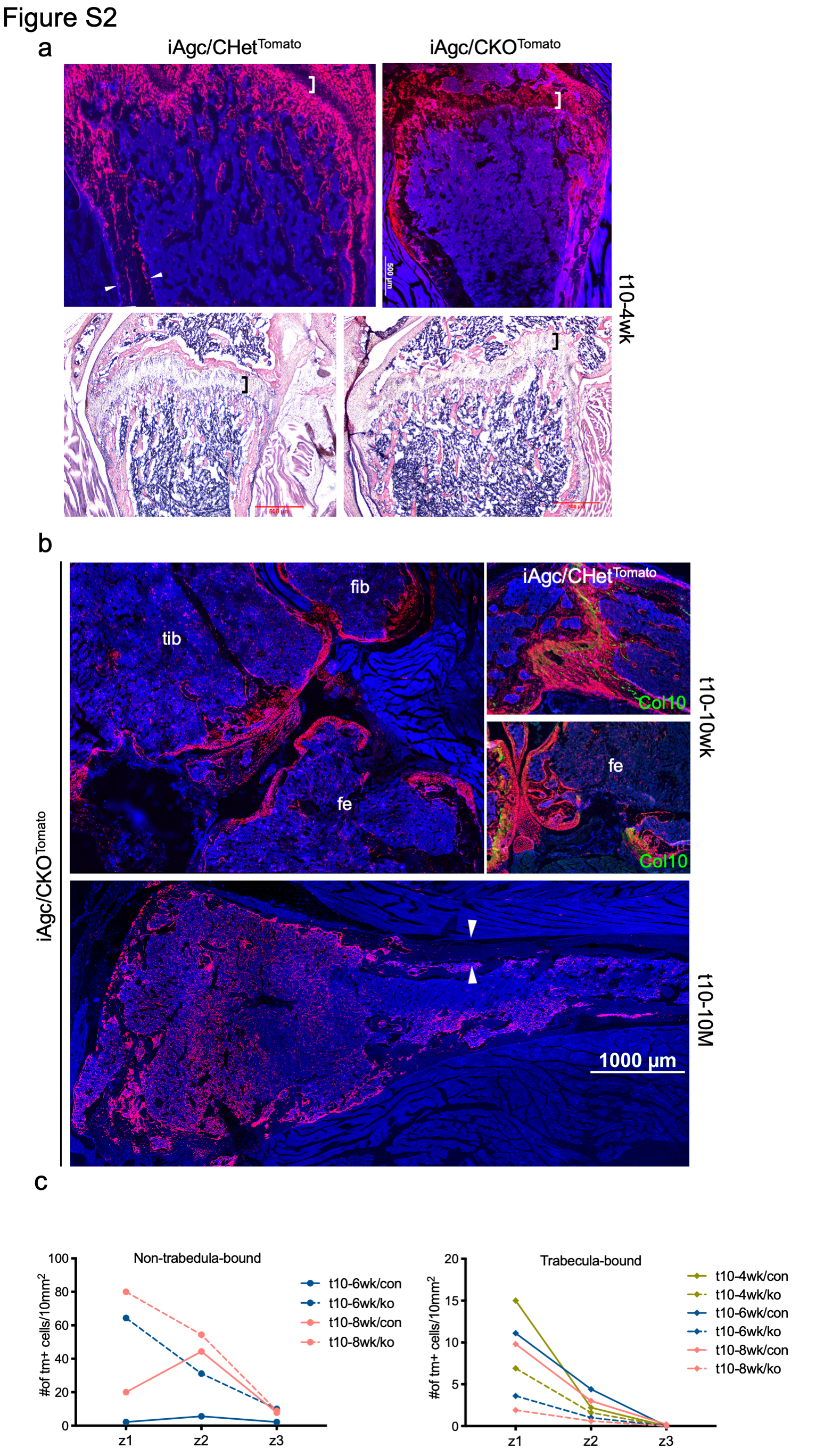

Supplement: Supplementary file 3 — Supplementary Figure S2 [file 41419_2021_3758_MOESM3_ESM.png]

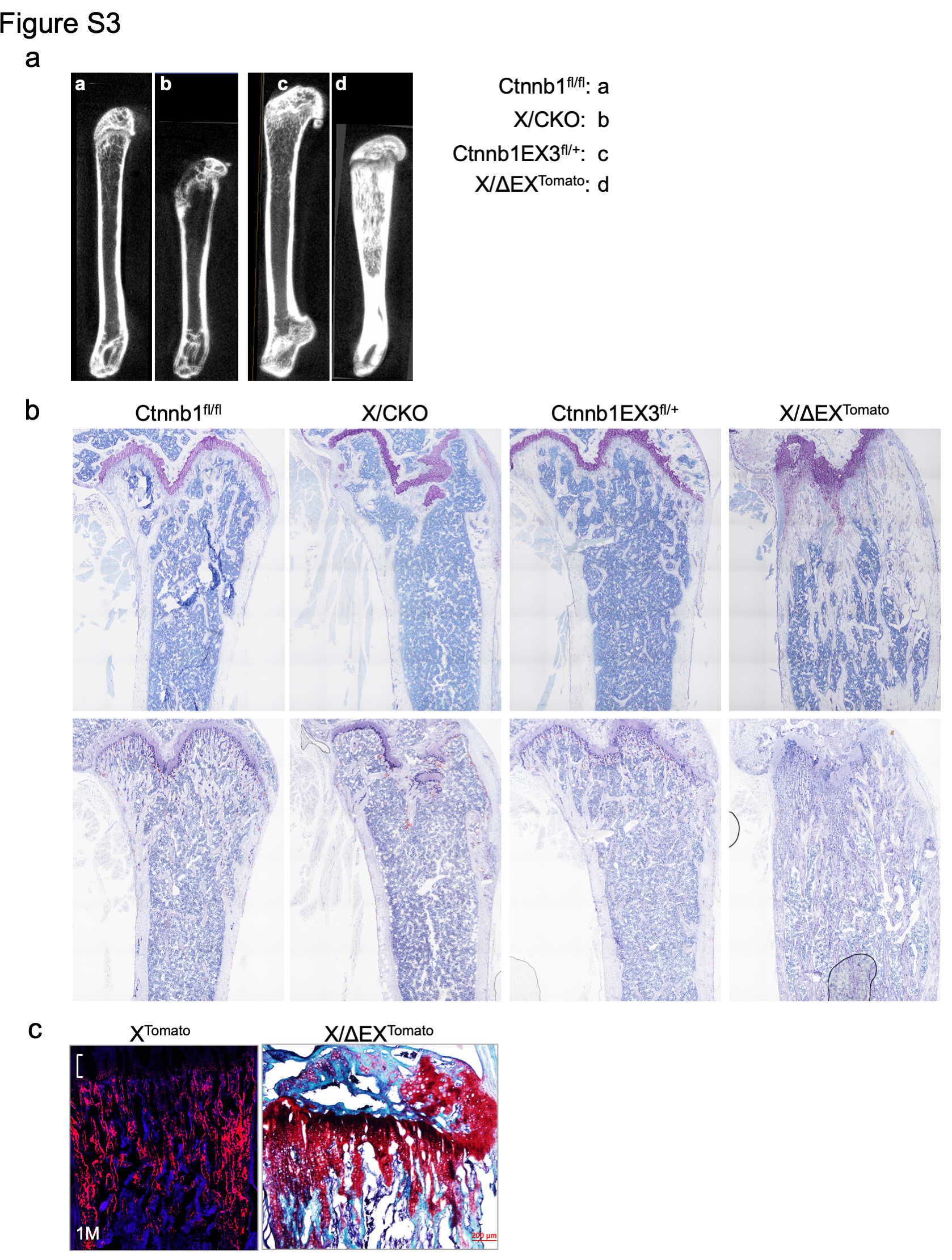

Supplement: Supplementary file 4 — Supplementary Figure S3 [file 41419_2021_3758_MOESM4_ESM.png]

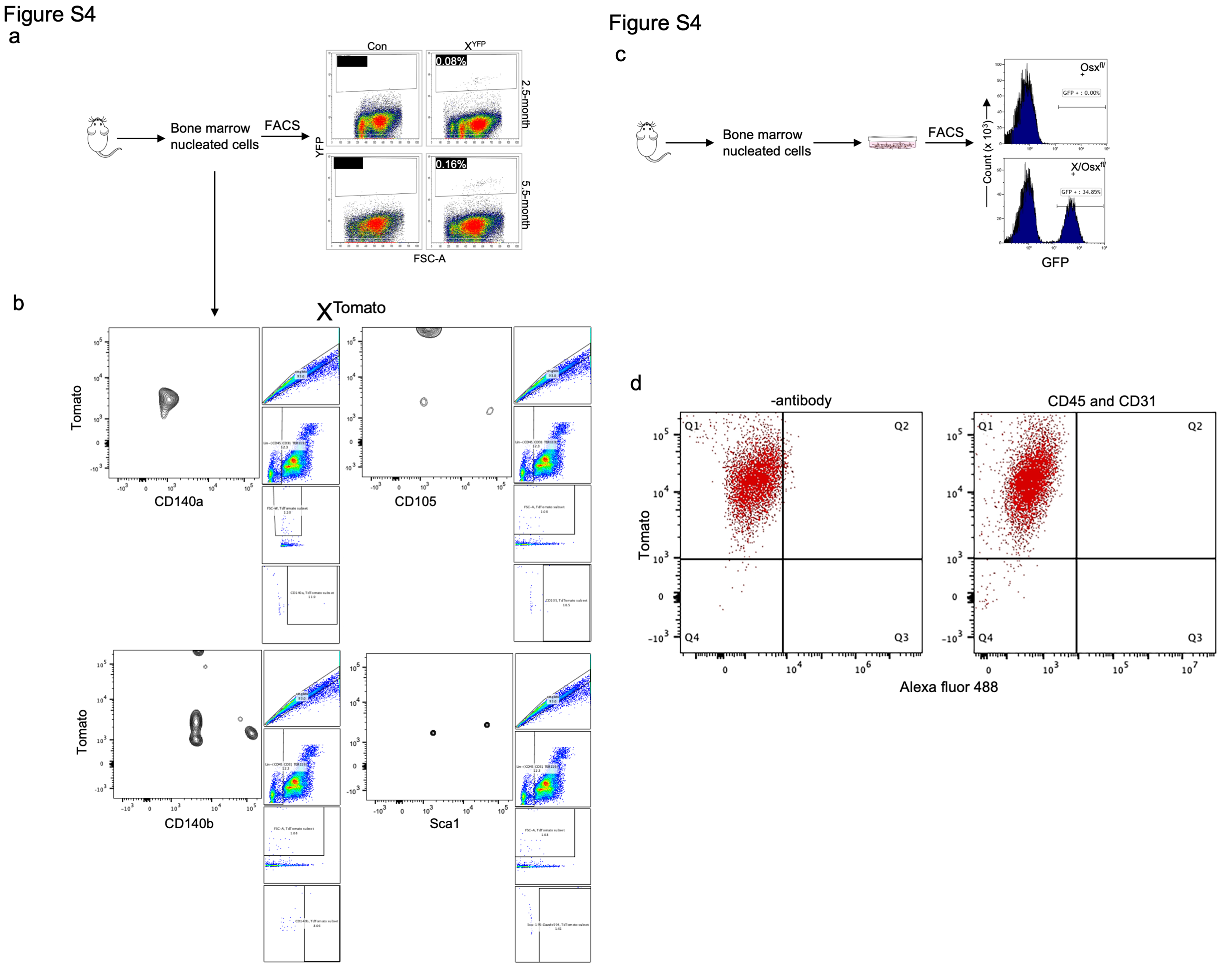

Supplement: Supplementary file 5 — Supplementary Figure S4 [file 41419_2021_3758_MOESM5_ESM.png]

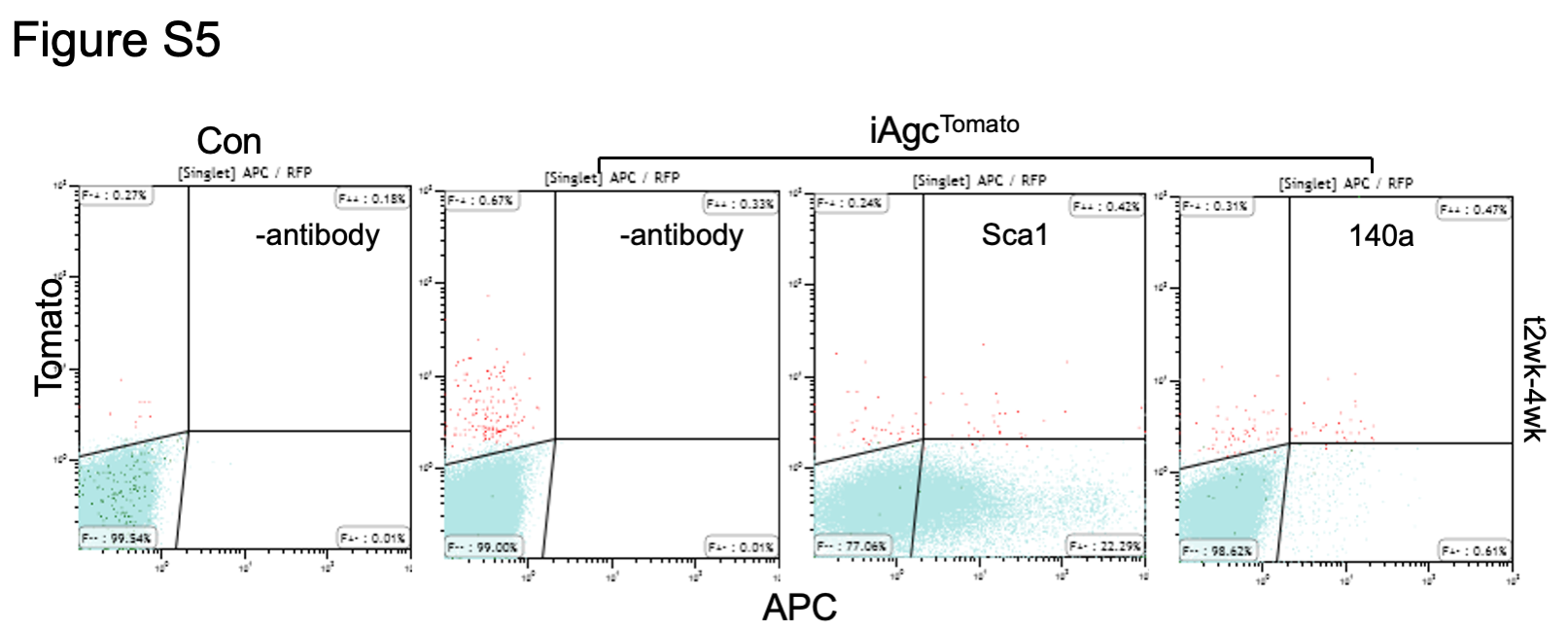

Supplement: Supplementary file 6 — Supplementary Figure S5 [file 41419_2021_3758_MOESM6_ESM.png]

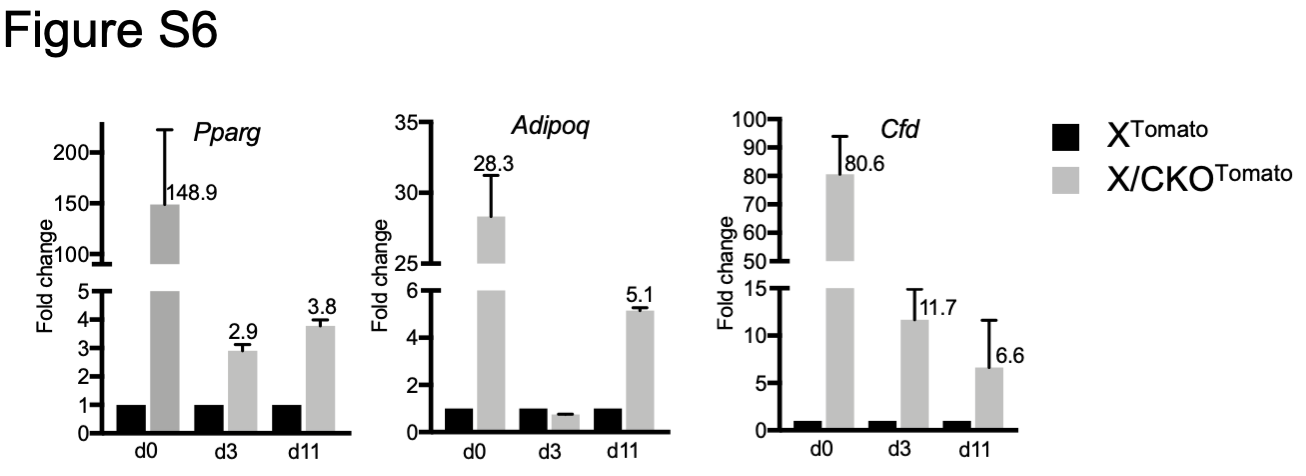

Supplement: Supplementary file 7 — Supplementary Figure S6 [file 41419_2021_3758_MOESM7_ESM.png]

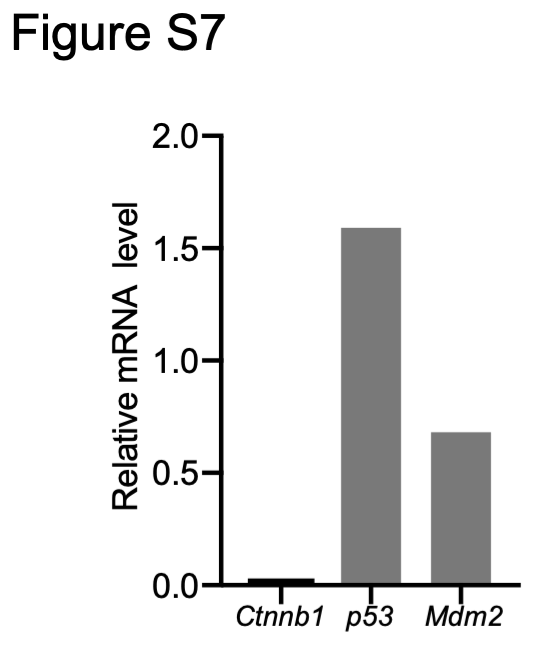

Supplement: Supplementary file 8 — Supplementary Figure S7 [file 41419_2021_3758_MOESM8_ESM.png]

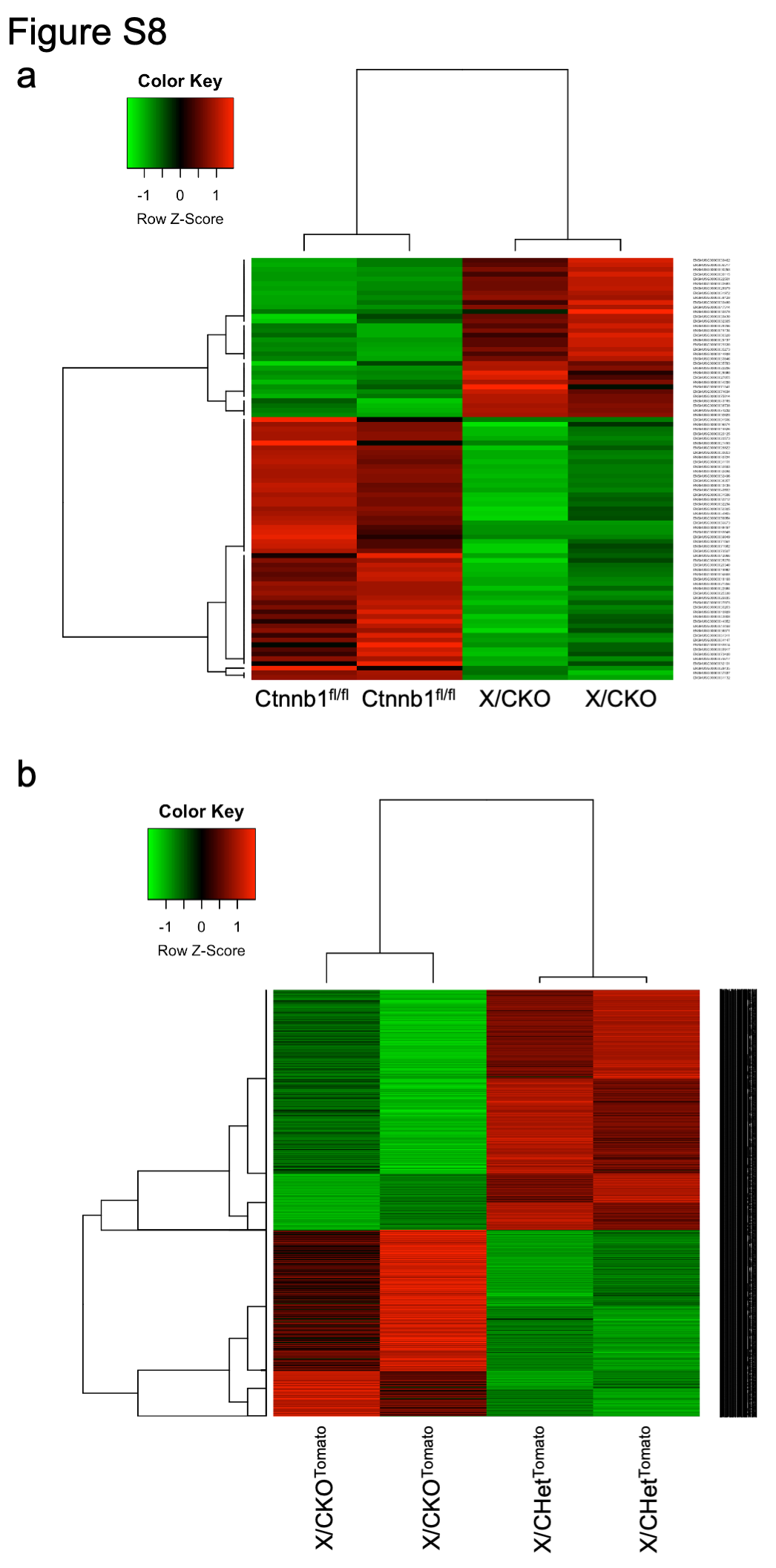

Supplement: Supplementary file 9 — Supplementary Figure S8 [file 41419_2021_3758_MOESM9_ESM.png]

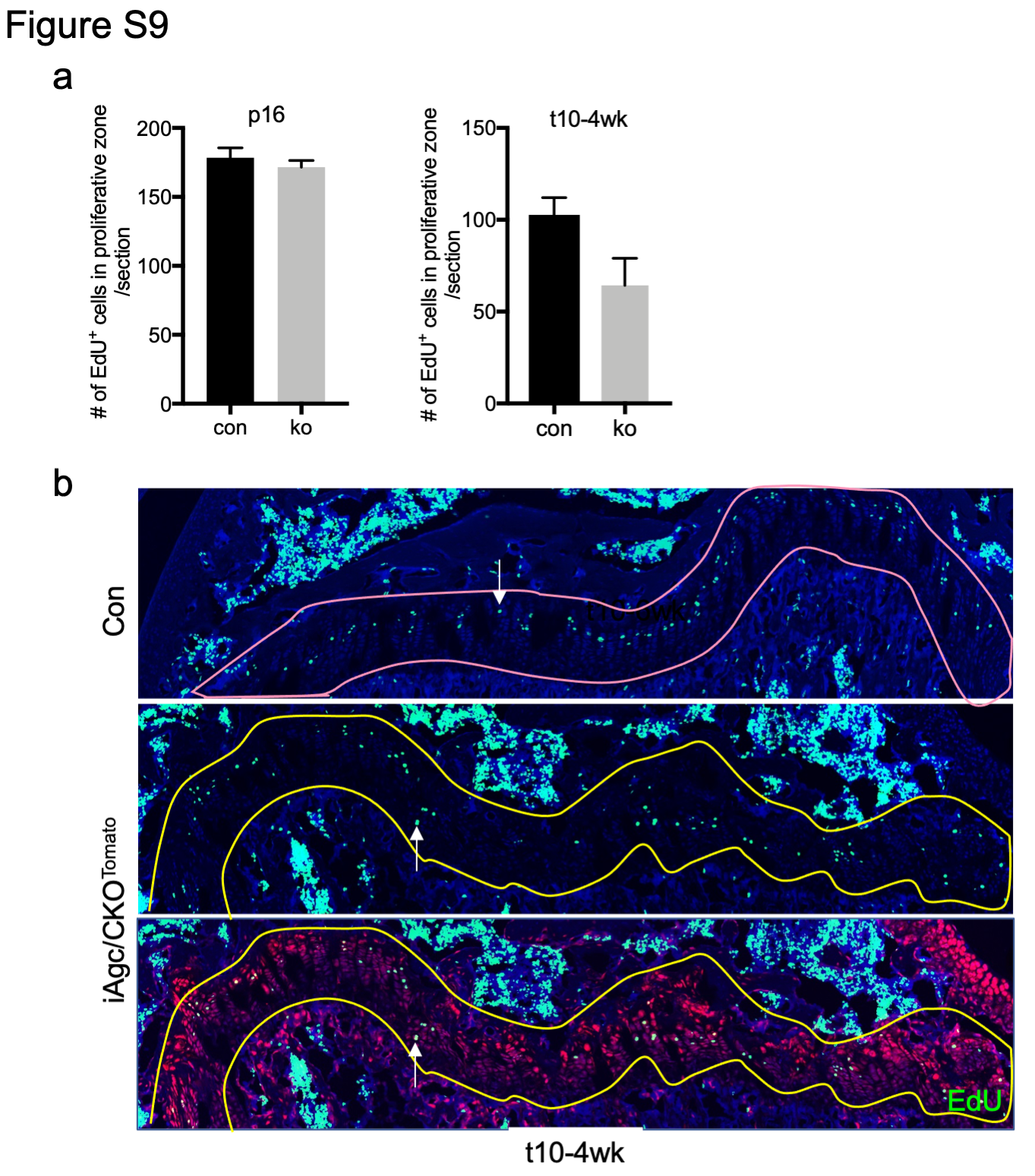

Supplement: Supplementary file 10 — Supplementary Figure S9 [file 41419_2021_3758_MOESM10_ESM.png]
